# Automatic Segmentation of Ultrasound Tomography Image

**DOI:** 10.1155/2017/2059036

**Published:** 2017-09-10

**Authors:** Shibin Wu, Shaode Yu, Ling Zhuang, Xinhua Wei, Mark Sak, Neb Duric, Jiani Hu, Yaoqin Xie

**Affiliations:** ^1^Institute of Biomedical and Health Engineering, Shenzhen Institutes of Advanced Technology, Chinese Academy of Sciences, 1068 Xueyuan Avenue, Shenzhen University Town, Shenzhen 518055, China; ^2^Shenzhen Colleges of Advanced Technology, University of Chinese Academy of Sciences, 1068 Xueyuan Avenue, Shenzhen University Town, Shenzhen 518055, China; ^3^Department of Oncology, The Karmanos Cancer Institute, Wayne State University, Detroit, MI 48201, USA; ^4^Department of Radiology, Guangzhou First Hospital, Guangzhou Medical University, Guangzhou 510180, China; ^5^Delphinus Medical Technologies, Inc., Plymouth, MI 48170, USA; ^6^Department of Radiology, Wayne State University, Detroit, MI 48201, USA

## Abstract

Ultrasound tomography (UST) image segmentation is fundamental in breast density estimation, medicine response analysis, and anatomical change quantification. Existing methods are time consuming and require massive manual interaction. To address these issues, an automatic algorithm based on GrabCut (AUGC) is proposed in this paper. The presented method designs automated GrabCut initialization for incomplete labeling and is sped up with multicore parallel programming. To verify performance, AUGC is applied to segment thirty-two in vivo UST volumetric images. The performance of AUGC is validated with breast overlapping metrics (Dice coefficient (*D*), Jaccard (*J*), and False positive (FP)) and time cost (TC). Furthermore, AUGC is compared to other methods, including Confidence Connected Region Growing (CCRG), watershed, and Active Contour based Curve Delineation (ACCD). Experimental results indicate that AUGC achieves the highest accuracy (*D* = 0.9275 and *J* = 0.8660 and FP = 0.0077) and takes on average about 4 seconds to process a volumetric image. It was said that AUGC benefits large-scale studies by using UST images for breast cancer screening and pathological quantification.

## 1. Introduction

Breast cancer threatens women's lives worldwide. It ranks as the second most common form of cancer with more than 1.3 million women diagnosed annually [1, 2]. In the United States, 12% of women will potentially develop this disease during their lifetime [1]. Consequently, breast cancer early detection is increasingly critical. Breast cancer screening plays an important role in early cancer detection, disease diagnosis, treatment planning, and therapeutic verification. In clinical applications, medical images serve as one of the primary means of breast cancer screening. Among the various modalities for breast cancer screening, mammography remains as the first choice, while supplementary modalities include hand-held ultrasound, computerized tomography (CT), and magnetic resonance imaging (MRI) [3, 4]. Of these commonly used modalities, mammography and hand-held ultrasound create two-dimensional (2D) images of the compressed breast, which leads to various deficiencies in clinical applications. Moreover, mammograms use X-ray imaging technology, exposing women to potentially harmful ionizing radiation. On the other hand, MRI provides three-dimensional (3D) images of the breast without exposure to ionizing radiation; however, its high cost prevents it from being widely adapted for breast cancer screening.

Practically, to promote an affordable and accurate 3D breast cancer screening imaging technique, Dr. Duric et al. developed a novel ultrasound tomography (UST) [5, 6]. It can scan the entire breast using ring array transducers with B-mode, which reduces breast compression and human subjectivity in image acquisition [7]. Moreover, no radiation is involved in UST imaging and breast anatomy is presented in 3D space [8]. As such, it aids in tumor differentiation in cases of obscured tumors or tumors located within dense breasts [5, 8–10]. In addition, UST volumetric images can be applied in breast density estimation [6, 11, 12], medicine response analysis [13], anatomical change, and breast tumor analysis [14, 15]. In summary, the image acquisition of UST is safe, cost-effective, and highly efficient. In clinical practice, breast segmentation affects follow-up image analysis for risk assessment, detection, and diagnosis [16, 17], as well as cancer treatment [18, 19]. Furthermore, extracting the breast region from surrounding water enhances tissue visualization and provides physicians and radiologist with superior understanding of breast tumor positioning [7].

To our knowledge, several algorithms have been developed toward UST image segmentation. Balic et al. [20] proposed an algorithm based on active contours, which is time consuming and its success depends on quality initial contour. Furthermore, this method is without a systematic evaluation of accuracy. Hopp et al. [21] presented a method integrating edge detection and surface fitting for breast segmentation. However, this method typically requires massive user interaction and postprocessing for outputting results. Sak et al. [22] took advantage of *K*-means [23] and the thresholding methods [24] to reduce user interaction, though proper parameters are yet needed in these methods. Generally, major methods suffer from heavy time consumption and excessive interactions not applicable to large-scale studies. In order to overcome these issues, we proposed an approach based on GrabCut for automatic segmentation of UST images. GrabCut utilizes incomplete labeling to reduce user interaction and seeks efficient segmentation in an iterative manner of energy minimization [25]. It falls under the graph cut method [26–31] and shows superiority in manual segmentation of 2D natural images, while in the presented algorithm, we provide an automatic approach for incomplete labeling of GrabCut, as well as deploying the algorithm on multicores to speed up the segmentation as demonstrated in [32].

The organization of this paper is as follows. The proposed method, experimental design, and evaluation criteria are presented in Section 2.  Section 3 presents experimental results from perceived evaluation to objective evaluation. Discussion and conclusion are given in Sections 4 and 5, respectively.

## 2. Methods

### 2.1. Proposed Algorithm

**Figure 1:**
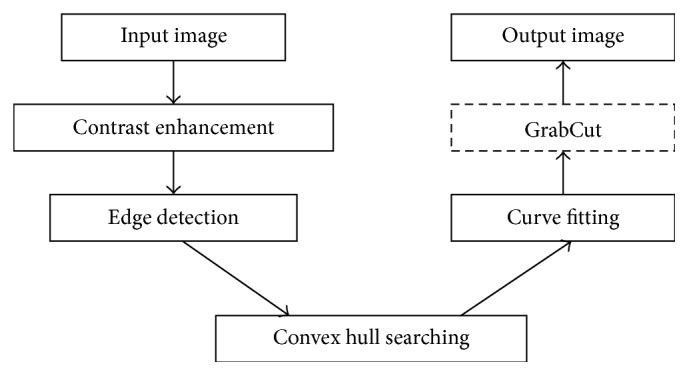
The flowchart of the proposed AUGC algorithm.

As a semiautomatic algorithm, GrabCut is widely used in various scenarios [33, 34]. However, in clinical applications, an automatic method that can lighten workload and minimize subjective bias is always desirable.  Figure 1 illustrates the proposed automatic method (AUGC) based on GrabCut. It integrates contrast enhancement, edge detection, convex hull searching, and curve fitting for automatic initialization of GrabCut.

**Figure 2:**
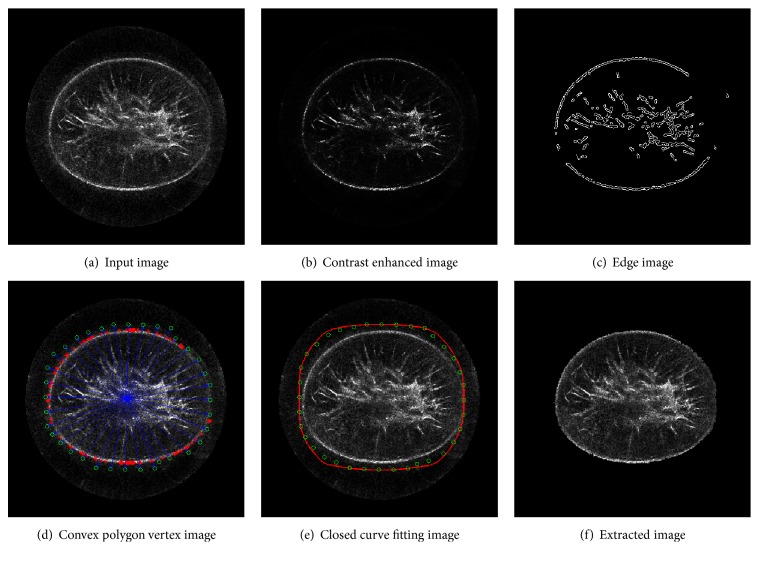
A case study of AUGC in UST image segmentation. (a) The UST image slice, (b) the input image after contrast enhancement, (c) the edge image detected by Canny with an adaptive thresholding, (d) the convex polygon vertex image produced by using convex hull searching and postprocessing, (e) the closed curve image produced by using Hermite cubic curve algorithm, and (f) the resultant image extracted by GrabCut. The figure can be enlarged to view details.

To make the flowchart clearer, we present a case study shown in Figure 2. First, after contrast enhancement, the input image suppresses image content in the background and highlights breast boundaries and glandular tissues (b). Then, the major boundaries are detected in (c). After that, convex hull searching is used, and key points (red sparkers) are found (d). Generally, the boundaries of the breast in UST images are not complete to a large extent. Taking robustness into consideration, we further push these key points outward (green circle) to enclose the tissue region of interest shown in (d). Next, green circles are interpolated with Hermite curve to form a bimap (red polygon) for incomplete labeling (e). In the end, (f) shows the extracted breast region.

*Image Preprocessing.* This step includes contrast enhancement and boundary detection of UST images. It uses sigmoid function to sharpen the image followed by a median filter with a kernel of [33] to suppress speckle noise. As shown in Figure 2(b), this step not only suppresses background image content, but also reduces speckle noise. Moreover, it benefits edge detection as shown in Figure 2(c), because image contrast is enhanced.

*Convex Hull Searching.* A fast convex hull algorithm is used to determine a convex hull point set *V*_hull_ = {*V*_*i*_}_*i*=1_^*n*^. With the point set *V*_hull_, we calculated a centroid *C* and a distance* R *between the farthest point *P*_*f*_ in the point set *V*_hull_ and *C*, as well as four extreme points of *x*_min_, *x*_max_, *y*_min_, and *y*_max_ for the regions of interest. A set of points *V*_psu_ = {*v*_*i*_}_*i*=1_^36^ are uniformly generated on the circle centered at *C* with the radius *R* (*R* = ‖*C* − *P*_*f*_‖_2_). Note that outliers in Figure 2(c) are removed with morphology operation before convex hull searching.

As shown in Figure 2(c), one problem occurs because the breast boundary is incomplete. To tackle this problem, the convex hull point set *V*_hull_ and the pseudo-contour point set *V*_psu_ are combined to refine the breast mask. The algorithm is given in Algorithm 1. 

Figure 2(d) shows the results after the convex hull searching and refinement, in which red sparkers are the original convex hull point set *V*_hull_, green circles are adjusted points for generating breast mask, and the blue point is the centroid *C*.

*Closed Curve Fitting.* Hermite cubic curve is powerful in smooth interpolation between control points [35] and four Hermite basis functions are described in

(1) h1s=2s3−3s2+1,h2s=−2s3+3s2,h3s=s3−2s2+s,h4s=s3−s2.

Moreover, the general form of Hermite curve is expressed in (2) below, where scale *s* goes from 0 to 1 with equal spacing Δ*s* (Δ*s* = 0.1). The closed curve in red is the Hermite curve with the inputting of the 36 points refined and shown in Figure 2(e). Pixels in the closed curve are the potential foreground, while outside of the curve is the definite background for GrabCut initialization.

(2) $$\begin{array}{c} P\left(\;s\;\right)=h_{1}\left(\;s\;\right)P_{0}+h_{2}\left(\;s\;\right)P_{1}+h_{3}\left(\;s\;\right)\mu_{0}+h_{4}\left(\;s\;\right)\mu_{1}. \end{array}$$

Here *P*_0_ and *P*_1_ represent the starting and the ending points of the curve, and *μ*_0_ and *μ*_1_ represent tangent to how the curve leaves the starting point and the ending point, respectively.

*GrabCut*. After closed curve fitting, Gaussian Mixture Models (GMMs) [31] are initialized with pixels inside and outside the closed curve and a flow network is built. In the network, each pixel represents a graph node. After that, a max-flow min-cut algorithm is applied for graph segmentation [27]. At last, a sample of the extracted breast is shown in Figure 2(f).

Overall, the procedure mentioned above handles only one slice in volumetric images and the entire breast UST volume is a stack of multiple gray-scale slices. As such, the proposed AUGC can be deployed with parallel programming as presented in [32]. Based on the advanced computer architecture, parallel programming can be realized on graphic processing units (GPUs) or on multicore central processing units (CPUs). GPU-based acceleration is difficult in algorithm development, in addition to costing extra programming time. On the other hand, parallel programming based on multicore CPUs is more promising as the technology is mature and comparatively easy to use. Personal computers with multicore CPUs are particularly easy to access; therefore, parallel programming based on multicore CPUs is utilized in the proposed method.

### 2.2. Algorithms for Comparison

Three algorithms are involved in this study. The first one, CCRG, utilizes simple statistics in region growth [36]. It calculates the median intensity *m* and the standard deviation *σ* based on a given region. A multiplier *l* should be supplied which defines a range around *m*. In our experiment, the multiplier *l* is adjusted to range from 3 to 5 times, the maximum iteration number is 500, and the seed radius is 4 mm.

The second algorithm, watershed, is a level set algorithm that classifies pixels into regions using gradient descent [37]. Additionally, a key parameter of watershed is water level (wl), tuned according to segmentation image. In our experiment, we start exploring it at 0.2. If too many small regions are obtained, we set it higher or else we tune it lower until a visually acceptable result is generated. Regarding different UST volumes, we found that water level ranges from 0.16 to 0.23. Since resultant regions are rendered by using different colors, a postprocessing step is used to merge these regions into two groups as the background and the breast region.

The final algorithm, ACCD, is derived from active contour evolution [38] and allows for control point delineation [39]. The number of control points is proportionally distributed to the region boundary length. Although basic active contour has more than ten tunable parameters, we focus on only two key parameters, *α* and *β*, which define the relative importance of the internal and external energy [38]. Note that we place control points near but not on the breast boundary. Compared to the original algorithm in [39], no refinement is involved.

At last, the classification and comparison of algorithms mentioned above were summarized in Table 1. For full knowledge of technical details, please refer to [35–39].

### 2.3. Case Study and Evaluation

*Data Collection*. Thirty-two whole-breast UST volume images are collected (SoftVue™, Delphinus Medical Technologies, Michigan, USA). The size of image slice is 512 × 512 and the physical resolution of UST volume is [0.5, 0.5, 2.0] mm^3^. An experienced radiologist defined the starting and ending slices following the procedure described in [14], and the average number of remaining slices in each volume is 17 ± 2. The radiologist also manually delineated the breast region in each slice to build the ground truth for algorithm validation.

*Software Platform.* AUGC and CCRG are implemented with VS2010 (https://www.visualstudio.com) in cooperation with OpenCV (http://opencv.org) and ITK (https://itk.org) [40], and ACCD is previously built with MATLAB [35], while the watershed algorithm is manipulated on VolView (https://www.kitware.com/opensource/volview.html). All codes are running on Windows 7 workstation with 4 Intel (R) Cores (TM) of 3.70 GHz and 8 GB DDR RAM.

*Accuracy Evaluation*. Three criteria, Dice (*D*), Jaccard (*J*) coefficients, and false positive (FP), are used to evaluate the accuracy of breast image segmentation [41]. These measures are defined in

(3) D=2G∩SG+S,J=G∩SG∪S,FP=S−G∩SG,

where *S* and *G* denote the segmentation result and the corresponding ground truth, respectively, while |·| denotes the breast voxel number. The values of these equations range from 0 to 1. Higher values indicate better performance for *D* and *J*, while a value of zero is achieved when performing perfect breast volume segmentation for FP.

To evaluate the real-time capability, time cost (TC) is defined as

(4) $$\begin{array}{c} TC=\frac{1}{t}\overset{n}{\underset{i=1}{\sum }}{tc}_{i}, \end{array}$$

where *t* is the number of total image slices, *n* is the number of breast volumes, and tc_*i*_ is the time cost for each volume. Note that time spent on parameters tuning and manual initialization for semiautomatic algorithms is not taken into account.

## 3. Results

### 3.1. Perceived Evaluation

**Figure 3:**
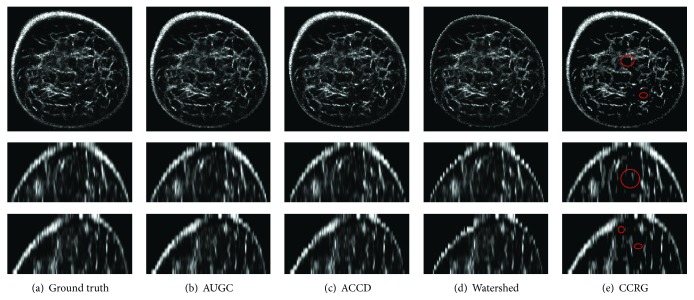
Perceived segmentation results of a UST image. (a) Ground truth produced by manual delineating, (b) AUGC, (c) ACCU, (d) watershed, and (e) CCRG. Images are interpolated in the sagittal and coronal view and then cropped in three views for display purpose. The figure can be enlarged to view details.

Perceived evaluation of segmentation results is shown in Figure 3. From left to right are the ground truth and resultant breast regions from AUGC, ACCD, watershed, and CCRG, while from top to bottom are the coronal, sagittal, and transverse view, respectively. Note that images are cropped for display purposes. No visual difference is observed between algorithms on this case, except that watershed fails to detect bright pixels on the breast boundary and CCRG fails to segment the foreground content shown in red circles.

### 3.2. Quantitative Evaluation

**Figure 4:**
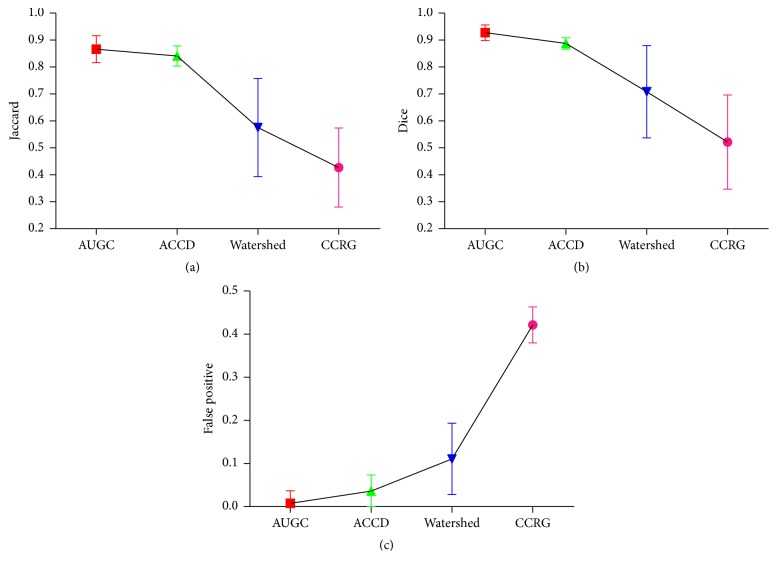
Accuracy evaluation of AUGC, ACCD, watershed, and CCRG segmentation methods, (a) represents the values of *D*, (b) is the values of *J*, and (c) denotes the values of FP. The figure can be enlarged to view details.

Quantitative evaluation of all algorithms for UST image segmentation is shown in Figure 4 where different colors indicate different algorithms. Moreover, (a), (b), and (c) represent the values of *D*, *J*, and FP, respectively. It indicates that AUGC outperforms other algorithms, followed by ACCD. Furthermore, both AUGC and ACCD feature relatively robust values of *D*, *J*, and FP.

### 3.3. Real-Time Capability

**Figure 5:**
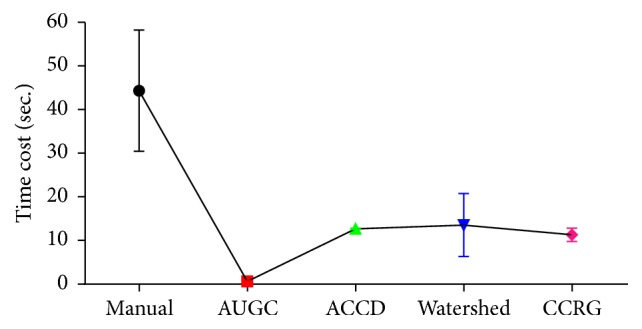
Real-time capability of involved algorithms. Time consumption is decreased dramatically from manual segmentation to AUGC. The figure can be enlarged to view details.

Time cost for each slice in UST image segmentation is shown in Figure 5. Compared to manually building the ground truth (44.33 seconds per slice), all the algorithms speed up the process of breast image segmentation. Particularly, AUGC dramatically shortens time consumption and makes it possible for real-time UST image breast segmentation.

### 3.4. Comprehensive Performance Evaluation

Table 2 illustrates overall performance of four algorithms. It reveals that AUGC achieves the best performance, not only providing the highest volume overlap measures (*D* and* J*), but also leading to the least error (FP). In addition, AUGC demonstrates the real-time capability in image segmentation. Inferior to AUGC is ACCD. Both watershed and CCRG achieve* J* value less than 0.6. Additionally, CCRG produces the lowest accuracy with the highest FP.

## 4. Discussion

UST holds tremendous promise for breast cancer screening and examination and UST images are preferred in clinical applications, such as quantitative breast tissues analysis [5, 9, 10], breast mass growing monitoring [6, 11], and clinical pathologic diagnosis [12–15]. In this paper, we presented a fully automated algorithm (AUGC) for breast UST image segmentation. The performance of four segmentation algorithms has been verified based on thirty-two volumetric images.

Quantitative evaluation of segmentation performance suggests that AUGC is superior to other three algorithms, ACCD, watershed, and CCRG, shown in Figures 3 and 4 and Table 2. Among these methods, CCRG resulted in the lowest accuracy and the highest amount of false positives. Moreover, watershed produced background content onto the final results. On the whole, ACCD is slightly inferior to AUGC. However, ACCD requires a user to locate several control points in the breast boundary. In addition, it contains more than ten parameters which need to be tuned manually, making the segmentation complicated and exhaustive. Generally, AUGC obtains the best performance in terms of segmentation accuracy.

AUGC is also superior to other approaches in terms of the real-time capacity. It can isolate an entire UST volumetric image within four seconds (0.2356 × 16 = 3.7696) on a four-core CPU system. Therefore, the greater the number of CPUs is, the less segmentation time it needs. It is known that real-time breast extraction plays a critical role in practical applications. For instance, breast density estimation is a routine task before rating breast cancer risk. At present, manual extraction of the whole breast in UST image hampers its large-scale experiments. Consequently, the proposed AUGC paves the way for large-scale studies in terms of high accuracy and real-time speed. It can accelerate the application of UST in anatomical change quantification, medicine response, and other related tasks.

The UST imaging technology is still under development and remarkable improvements have been made recently [42, 43]. These improved technologies are bound to enhance UST image quality and tissue contrast. High UST image quality can improve the performance of AUGC in breast segmentation, suggesting an even greater potential of AUGC to facilitate clinical diagnosis by using whole-breast UST images

## 5. Conclusion

UST image segmentation not only is time consuming, but also requires massive user interaction. An automated algorithm based on GrabCut is proposed and verified in this study. Experimental results have validated its good performance in UST image segmentation. Furthermore, it can segment one slice within less than 0.3 seconds. It is beneficial for large-scale studies and physicians can also be released from the tedious task of UST image segmentation.

## Figures and Tables

**Algorithm 1:**
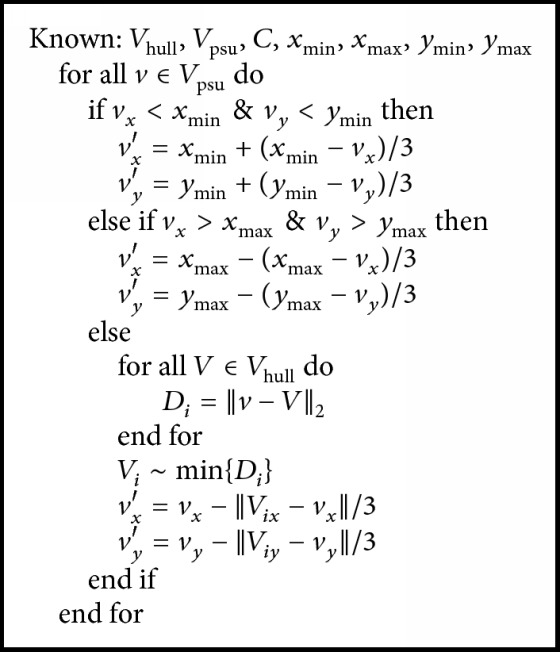
Convex hull refinement (the refinement flowchart of the convex hull points).

**Table 1:** Classification and comparison of involved algorithms in this paper.

	Category	Initialization	Tuned parameters
AUGC	GrabCut	Canny operator with adaptive thresholding and the structure element radii of morphological operators is initialized to 4 and 8	—

ACCD	Active contour	20 control points per slice	*α* and *β*

Watershed	Level sets	0.1	wl

CCRG	Region growing	10 seeds for each volume	*l*

**Table 2:** Comprehensive performance evaluation of involved algorithms.

	Dice (*D*)	Jaccard (*J*)	False positive (FP)	Time cost (TC)
AUGC	0.9275	0.8660	0.0077	0.2356
ACCD	0.8874	0.8407	0.0362	12.6742
Watershed	0.7084	0.5757	0.1107	13.5360
CCRG	0.5218	0.4268	0.4214	11.3120
