# Erratum to “Automatic Segmentation of Ultrasound Tomography Image”

**DOI:** 10.1155/2017/6258395

**Published:** 2017-11-05

**Authors:** Shibin Wu, Shaode Yu, Ling Zhuang, Xinhua Wei, Mark Sak, Neb Duric, Jiani Hu, Yaoqin Xie

**Affiliations:** ^1^Institute of Biomedical and Health Engineering, Shenzhen Institutes of Advanced Technology, Chinese Academy of Sciences, 1068 Xueyuan Avenue, Shenzhen University Town, Shenzhen 518055, China; ^2^Shenzhen Colleges of Advanced Technology, University of Chinese Academy of Sciences, 1068 Xueyuan Avenue, Shenzhen University Town, Shenzhen 518055, China; ^3^Department of Radiation Oncology, Northwestern Medicine Lake Forest Hospital, Lake Forest, IL 60045, USA; ^4^Department of Radiology, Guangzhou First Hospital, Guangzhou Medical University, Guangzhou 510180, China; ^5^Department of Oncology, The Karmanos Cancer Institute, Wayne State University, Detroit, MI 48201, USA; ^6^Delphinus Medical Technologies, Inc., Plymouth, MI 48170, USA; ^7^Department of Radiology, Wayne State University, Detroit, MI 48201, USA

In the article titled “Automatic Segmentation of Ultrasound Tomography Image” [1], the affiliation of the third author was incorrect. The corrected authors' list and affiliations are shown above.
